# Sea Travel without Sea Sickness

**Published:** 1896-07-11

**Authors:** 


### Sea Travel without Sea Sickness.

In England, where the larger portion of the population reside in inland towns, individual families not unnaturally consider that the best form of holiday, conferring the maximum of health benefits, is to be had at the seaside. Every year an increasing number of people spend some weeks at a seaside resort. Indeed, it is becoming increasingly difficult to find any rural seaside resort in these islands, seeing that the demand for accommodation has so vastly grown everywhere that buildings have multiplied and rural populations have been gradually overborne by the influx of new residents who compete with each other in making provision for the holiday population every season. Very many Englishmen and Englishwomen would gladly take a sea voyage during a portion at any rate of their holiday did they not dread *mal de mer*. It seems, however, by no means improbable that by far the greater majority might easily, if they chose, protect themselves from sea-sickness and the unpleasant results of the functional disturbance of the nervous system produced by the motion of a ship. It is undoubtedly true that a large proportion of those who take their holidays on the Continent begin to be sea-sick from the time the train leaves the London terminus on its way to the coast town from which the steam packet starts. Anyone who cares to go to Victoria Station and enter a carriage in the tidal express may easily discover several persons at least lying down at their full length with white faces and troubled aspect, who are already experiencing *mal de mer*. It would be wrong to denounce the sufferers as hysterical or idiots. They merely lack self-discipline, and have not resolution enough to exercise common sense and to refuse to be ill without good cause, and then only after a tough fight to overcome the strain upon their system produced by the motion of a vessel upon a stormy sea. There is but little doubt, however, that could they be taken in hand individually two or three days before entering a train, they could be brought to realise the absurdity of the attitude they assume in regard to sea-sickness and sea travel.

Those who go down to the sea in ships, especially if they be landsmen desirous of avoiding *mal de mer*, must prepare themselves for their contest with the sea by regular living and by a continuance of that regularity up to the time they go on board the ship, and during the whole period they may remain on it. There seems in fact every reason to believe that it is not difficult to acquire the habit which will enable the nervous system of a healthy person to adapt itself readily to the new condition of motion, and to overcome effectually the disturbing influence of the movements of the ship. There are people of much experience who consider that any person who boldly stands up in the wind's eye, and allows the body to swing with the movements of the ship itself, can in the course of a couple of hours or less acquire the habit referred to, and obtain freedom from sea-sickness during the sea voyage, no matter how rough the passage may be. Half a wineglassful of seawater, taken as soon as the motion of the ship becomes unpleasant, is thought by some not to produce, but actually to prevent, sea sickness in persons who are most susceptible under ordinary conditions to its influence. It has also often been noted that in the case of women especially, who are very liable to *mal de mer*, chlorobrom is an effectual remedy. Chlorobrom is a solution of chloralamid and bromide of potassium. It is stated by those who have watched its effects that it can be taken without risk of any kind, and that a full dose taken by the passenger, who should at once retire to his cabin to rest and sleep, will effectually prevent sea-sickness. It follows from what has already beeu stated that the most timid person, if properly advised by a competent medical practitioner, who prepares for the voyage by taking a couple of blue pills overnight, and who resolutely schools himself not to resist, but to accommodate the motions of his body to the motions of the vessel, may overcome sea-sickness, and enter into the full enjoyment of a sea voyage, by devoting the first few hours of the journey to the acquirement of the habit which enables the nervous system to adapt itself to the motions of the ship.

No doubt some readers, at any rate, will smile with unbelief when they read such views on the subject of sea-sickness. It may therefore be pointed out to them, and to all who are fond of the sea in theory or in fact, that the conditions of travel now provide a means of continuous journeying on the sea without any risk of sea-sickness of any kind. The Norwegian cruises, lasting from fourteen days to one month, may be confidently entered upon without any fear of sea-sickness except only during the few hours of open sea between this country and Norway. At the present season of the year and during the summer it is unusual to experience any sea of consequence in crossing from Leith or any southern port to Norway. But even this risk may be avoided by going to Stockholm mostly overland, and proceeding thence viâ Christiania to join the pleasure yacht at Bergen. From Bergen to the North Cape, a distance of some twelve hundred miles, the ship will continuously proceed through ever-changing and ever-beautiful mountainous scenery of very varying aspect, diversified by waterfalls and beautified by the skill with which the Norwegian cultivates every bit of soil which offers the slightest resting-place for crops of any kind. Passing continuously through salt water under the most favourable conditions, without any perceptible motion, so far as the ship is concerned, sea travel may be enjoyed to the utmost by the most squeamish of sailors, as any possibility of *mal de mer* in any form is rendered practically impossible from the circumstance that the Atlantic Ocean is effectually barricaded out by the Skjaergaard island belt or natural sea wall, which affords admirable shelter to the steamers and protects nearly the whole of the Scandinavian coast from Vads to Haparanda. It is impossible for anyone who has not made the voyage to realise the wonderful protection afforded by this island belt, which is so effectual as to make the water in the fjord perfectly calm, no matter how high the seas may be in the adjacent Atlantic Ocean. These Norwegian cruises, which have been so thoughtfully placed within the means of the majority of the middle classes of this country, should be readily taken advantage of by all who desire to extend their mind and knowledge, and to secure the blessings of improved health for themselves or their families.

